# Experience of the Egyptian Physical Therapy Educators on the Online Teaching During COVID-19 Outbreak 2021

**DOI:** 10.1080/10872981.2022.2073861

**Published:** 2022-05-13

**Authors:** Salwa B. El-Sobkey

**Affiliations:** Associate Professor of Physical Therapy for Cardiopulmonary Disorders and Geriatrics, Faculty of Physical Therapy, Beni-Suef University, Beni Suef, Egypt

**Keywords:** Egyptian Physical Therapy Educators, online teaching usefulness, COVID-19, practical and clinical instructions, social negative effect, financial overwhelming, entry-level physical therapy

## Abstract

Physical distance was one of the safety measures that were applied during the outbreak of COVID-19 and universities all over the world were forced to shift toward online teaching (OT). The aim of the study was to answer six research questions related to the profile of OT in Egyptian Physical Therapy Colleges during the COVID-19 outbreak. A google form questionnaire was used to survey 102 Egyptian Physical Therapy Educators (EPTEs) who were engaged in teaching Physical Therapy undergraduate programs in Egyptian universities during the spring semester of the academic year 2020-2021. Results showed that the EPTEs frequently (N= 51; 58.0%) used OT both from work and home. Private universities showed a significant advantage over public universities regarding the provision of institutional training (N= 101, P= 0.003) and availability of institutional educational support centers (N=99, P= 0.0001). Most (N= 30; 63.8%) university website users were full or associate professors, while (N= 24; 53.3%) Microsoft Teams users were lecturers. The EPTEs who had a positive attitude toward the suitability of OT for practical and clinical instructions were a minority (N= 48; 22.9%) and (N= 24; 29.2%) respectively. The EPTEs perceived different themes for advantages, disadvantages, and challenges regarding their experience with OT. Less than one-tenth (N= 10; 9%) of EPTEs showed the highest positive attitude toward the readiness of their colleges for the application of OT. Most (N= 68; 68%) of the EPTEs reported the presence of negative effects on their social life and (N= 30; 30%) of them reported high levels of financial overwhelming. In conclusion, the EPTEs had a limited and primitive profile of experience with OT during the COVID-19 outbreak in 2021. OT might not be the perfect teaching approach for Egyptian Physical therapy Colleges, especially for practical and clinical courses .                       .

## Background

The coronavirus disease was identified in Wuhan, China, in December 2019 (COVID-19) [1]. The World Health Organization announced a global outbreak in January 2020 [2] and physical distance, for safety, was applied by countries worldwide [3,4]. Authorities restricted citizen gathering and closed many institutions including the universities [2,5], which were confronted with immediate transformation into online learning [5]. Consequently, university educators found themselves in a situation of mandatory alteration with almost no choice and no time for preparation [2,6]. Online teaching (OT) was a challenge for both educators and students [5] and the sudden emergence of OT caused a hard situation for universities, especially those in developing countries, because of insufficient preparation [6]. During the spring semester of the academic year 2019–2020, Egyptian universities applied closure and transformation into OT. Year after, in the spring semester, the academic year 2020–2021 was the second shutdown of the Egyptian universities, and this time the Egyptian ministry of higher education delegated the decision for the universities and colleges to apply either OT or blended teaching according to the appropriateness to their academic programs.

While OT instructions are delivered to the students through the internet, blended teaching instructions are partly delivered through the internet and partly through face-to-face classes [7–10]. The OT includes two application approaches: the first approach is synchronous in which classes take place in real-time and connect instructors and all students via streaming audio or video or through a chat room and the second approach is asynchronous in which students can log on to and work on at their own time even if no one else is logged on at that time [7,10]. It is to be noted that advancements in communication technologies such as social networks, web-based resources, and discussion boards fostered the growth of OT globally [11,12], and even before the COVID-19 outbreak Egypt has been developing, since 1985, its infrastructure for communication technology and continuously it promotes it in higher education to motivates educators and students toward OT [13]. Add to that, many Egyptian universities invested in technology and used OT [14,15] to attract students, enhance the internationalization of education, fulfill customers’ needs, and overcome problems like large capacities of classes, high prices of traditional educational books, and transportation problems [14–16]. Meanwhile, a debate is existing regarding the involvement of OT in health professionals’ education, and support for claimed benefits of this involvement is opposed by some advice for caution and moderation [12]. Benefits include improvement of the communication network, enhancement of learner experience personalization, supporting of the life-long learning and assuring of quality content as well as allowing flexibility of access, enhancing practical skills performance and knowledge acquisition, enhancing deep learning, and encouraging reflection [11,17,18]. On the other hand, those opposing this involvement disagree with the students’ inclusion in a learning environment with an online learning process in which they are far from their educators and peers, and they are interacting with an OT system mediated by technology, which might influence the students’ engagement and questions the acquisition of skills and even knowledge [6,11,19,20].

In Egypt, the PT is a vital health-care profession that emphasizes the use of Physical therapy approaches in the promotion, maintenance, and restoration of an individual’s physical, psychological, and social well-being [21]. The urgent need for physical therapy services was recognized after the tripartite invasion of Egypt in 1956, with the need to rehabilitate injured civilians and military personnel [21]. The PT practice in Egypt is supported by evidence of clinical effectiveness and practitioners may work independently or as members of the health-care team in different health-care facilities [21]. The bachelor’s degree is the entry-level practice and its curriculum that lasts for 5 years and 1 year of internship, transfers knowledge, skills about patient assessment, treatment intervention, treatment outcomes, clinical analysis, discharge planning, and follow-up [21]. To this study date, reviewing literature revealed that studies regarding OT in health professions are directed more toward dental, nursing, and medical programs [16,17,20,22] and there is a lack of focus on PT programs. Also, studies are mainly concerned with the students’ perception, not the PT educators. Add to that, implementation of OT in the Egyptian educational system is recent and there are limited available studies [6,9,14,15,18,23,24] and they either focused on the student population, not including PT, or focused on information technology institutions. An example of the studies conducted on the students is the commentary done by Rossettini et al., in which they concluded that the PT students were motivated by the OT to overcome geographical barriers, but at the same time, the students were not sure about achieving the learning outcomes of a clinical case-based course and they perceived limitations the OT in terms of practical and clinical instructions, both for teaching and assessment [25]. Another study compared the satisfaction of one course between two groups of Italian PT students (46 OT and 112 face-to-face) and it revealed that the OT may be a feasible option to face the COVID-19 [26]. Internationally, there are a few studies that investigated the perception of PTEs regarding OT as the study done by Plummer in Brazil, the USA, and Cyprus [2] but to the author’s knowledge, there is no Egyptian study that highlighted the OT for PT program and at the same time focused on PT educators. That is why this study aimed to identify the OT experience of Egyptian Physical therapy Educators (EPTEs) during the COVID-19 outbreak in the spring semester of the academic year 2020–2021. The study scope is limited to undergraduate PT programs and is concerned with answering the following six research questions: 1 – How was EPTEs’ OT profile during the COVID-19 outbreak in 2021? 2 – What were the characteristics of EPTEs and their institutions that influenced this profile? 3 – How did EPTEs perceive the usefulness of OT for PT colleges, advantages, and disadvantages? 4 – To which level were Egyptian PT colleges and educators ready to implement OT and what were the challenges? 5 – Was there any social or financial implication of OT from home on EPTEs? 6 – How was EPTEs’ overall satisfaction with OT and what were the implicated factors? Answering these questions would help policymakers to figure out OT status in Egypt and it would provide knowledge that can be used as a base for strategic management of OT in Egypt.

## Methods

The checklist for reporting results of internet e-surveys (CHERRIES) was used to report the methods section of the current study [27,38].

## Survey design

This study used a structured, literature-based questionnaire [4,6,7,11,14,16–20,24,28–30] to collect the relevant data related to the study research questions. The questionnaire was designed in the English language and was composed of an introduction and three sections that included 33 items informed of quantitative (closed-ended and Likert-type) as well as qualitative (open-ended) question items. The introduction included the welcoming of the participants, study aim and scope, procedural definitions (OT, blended teaching, and e-course), an explanation of how to use the Likert rating scale (1–5), and the average duration for answering. The first section was data about EPTEs, the second section was regarding the description of courses and the third section was details about participants’ experiences with OT.

## IRB (Institutional Review Board) approval and informed consent process

The participants in this study were PT educators, not patients, and the need for ethics approval was waived and was deemed unnecessary by the internal research committee of the Physical Therapy College at Beni-Suef University. In the introduction of the questionnaire, it was explained to the potential participants that their response to the questionnaire is considered consent to use their data for the research purpose.

## Data protection

The collected data were entered in an excel sheet on the author’s laptop and was protected using antivirus software and a firewall.

## Development and testing

During the first 2 weeks of August 2021, pilot study was conducted in which a word document of the initial version of the questionnaire was sent (WhatsApp) to nine EPTEs of different ranks from different universities known to the author with objectivity, honesty, and commitment, and they were invited to respond to the questionnaire, report about (1) the convenience of the questionnaire’s items to the aim, and title of the study (content validity); (2) the time consumed to complete the questionnaire; and (3) the presence of unclear or redundant questions, or any ambiguous words. The nine EPTEs reported the content validity of the questionnaire, on average 20 minutes are required to respond to the questionnaire, and they recommended one question to be modified and two words to be replaced for more clarification. The author applied the recommended changes and prepared an electronic final version of the questionnaire as a Google form (available at https://forms.gle/p5sh7gittg1aWKmq8). The author invited the same nine PT educators who participated in the pilot study to respond to the electronic version and report on its usability and technical functionality before fielding it.

## Recruitment process

The author did not use any advertising for the survey and the recruitment process used the closed-survey approach in which only the invited potential participants were able to access the survey through its URL link, which was sent to them through social and professional applications like WhatsApp, professional and personal emails, and messenger. The recruitment process was based on eligible criteria. The inclusion criteria were as follows: EPETs who were engaged in teaching undergraduate programs in Egyptian universities during the spring semester – the academic year 2020–2021. The exclusion criteria were as follows: (1) EPTEs working for other universities outside Egypt, (2) EPTEs on sabbatical leave, (3) female EPTEs on maternity leave, (4) EPTEs in spouse’s accompany leave or sick leave, (5) EPTEs only engaged in the teaching of postgraduate programs, (6) EPTEs had no teaching load in the spring semester – the academic year 2020–2021. Egyptian universities that include PT colleges are 23 (6 public and 17 private universities) and according to available data on colleges’ webpages during the study time, the total number of EPTEs was 1070. According to the author’s more than 25 years long experience in the field of PT education, the sample frame was taking into consideration that about 20–25% of the EPTEs are working or studying abroad, 15–20% are emirate professors teaching only the post-graduate programs, 15–20% had no teaching load in spring 2021, and 10–12% were on leave of different causes and consequently out of the total number of 1070 EPTEs, the estimated number of the potential participants was about 40–45%, 460 EPTEs. Online power analysis (available at Sample Size Calculator (Use in 60 Seconds) // Qualtrics) [31] was used to calculate the study sample size. With a target population of 460, a confidence interval of 95%, and a margin error of 10%, the ideal sample size was 80 EPTEs.

## Survey administration

There were no incentives introduced to the participants who voluntarily participated in the study. During the last 2 weeks of August and the first week of September 2021, the Google form URL was sent to the potential participants. The EPTEs from the 23 PT colleges were invited to participate in the study except for the PT college at Suez Canal University because it is included in the list of Egyptian universities with PT colleges, but the college was not commenced yet.

During the second and third weeks of September 2021, two kind reminders were sent to un respondents to encourage them to participate in the study. The reminders were sent through professional and personal emails, messenger, and WhatsApp. During these 5 weeks (August 16–September 21, 2021), the author repeated the cycle of sending a new invitation for participation and reminders for previous invitations and applied convenience sampling to reach the number of 80 participants as calculated by the online sample size. The author used adaptive questioning in which the participants can skip and move to the next specific items if their answers were no to a current item to reduce the number and complexity of the questions. There was no use of mandatory items or randomization of items or questionnaires. Also, there were no completeness checks before the questionnaire is submitted. The author was dependent more on the commitment of the PT educators and preferred freedom and flexibility to the potential participants. The potential participants were able to review and change their answers through a back button.

## Completion rate

The author invited 325 EPTEs to participate in the study and received a positive reply for participation from 110 educators while the number of educators who submitted the questionnaire was 102. The completion rate in terms of the number of educators who submitted the questionnaire divided by the number of those who agreed to participate is 92.7%.

## Analysis

The quantitative responses of the participants to closed-ended questions of the questionnaire were coded and tabulated in the Excel sheet and then entered the program for statistical analysis of sampled data PSPP. Descriptive and inferential statistical tests were used to answer the research questions of the study considering a significant P-value less than 0.05. Results were presented in tables and figures. The qualitative responses of the participants to the open-ended questions of the questionnaire were recorded on an excel sheet, reviewed carefully and thoroughly, and categorized into themes and sub-themes items. The frequencies of each subtheme were calculated and represented in terms of frequency. Both the completed questionnaire and those with some missing data were involved in the data analysis. For quantitative data analysis, the size of the missed data was identified, and statistical analysis was applied to the completed data.

## Results

The calculated sample size was 80 EPTEs, the participants’ EPTEs were 102 and their descriptive characteristics are presented in Tables 1 and 2. Results are categorized into six sections in line with the six research questions:

**Table 1.** Egyptian Physical Therapy Educators’ rank, subspecialties, years of experience, and their colleges’ years of Commence

Rank	Frequency		Valid (%)		Physical therapy subspecialty			Frequency	Valid (%)
Lecturer	40		40.4		Basic Sciences and Biomechanics			30	30.4
Full or associate professor	22		22.2		Cardiopulmonary Disorders and Geriatrics			21	21.0
Assistant lecturer	17		17.2		Pediatrics			19	19.0
Current or former top management rank^a^	16		16.2		Musculoskeletal Disorders			10	10.0
Demonstrator	4		4.0		Integumentary			8	8.0
Total responses	99		100.0		Neurology			8	8.0
Missing data	3		2.9		Women’s Health			4	4.0
					Total Responses			100	100.0
					Missing Data			2	2.0
^a^Dean, Vice-dean, Department chair									
Participants’ experience (Years)^a^		Frequency		Valid(%)		Participants- colleges’ commence (Years)^b^	Frequency		Valid (%)
1–9		52		52.0		1–9	59		59.5
10–19		24		24.0		10–19	3		3.0
20–29		17		17.0		20–29	13		13.1
30–39		5		5.0		30–39	0		0.0
40–49		2		2.0		40–49	0		0.0
Total responses		100		100.0		50–60	24		24.2
Missing data		2		2.0		Total responses	99		100.0
						Missing data	3		2.9
^a^Minimum = 1 year, Maximum = 40 years, Mode = 6 years						^b^Minimum = 1 year, Maximum = 59 years, Mode = 8 years

**Table 2.** University sector and previous COVID-19 disease of the Egyptian Physical Therapy Educators

University sector	Frequency	Valid (%)	Previous disease with COVID-19	Frequency	Valid (%)
Public	51	50.5	Not diseased	58	58.0
Private	50	49.5	Diseased	42	42.0
Total responses	101	100.0	Total responses	100	100.0
Missing data	1	1.0	Missing data	2	2.0

**Figure 1.**
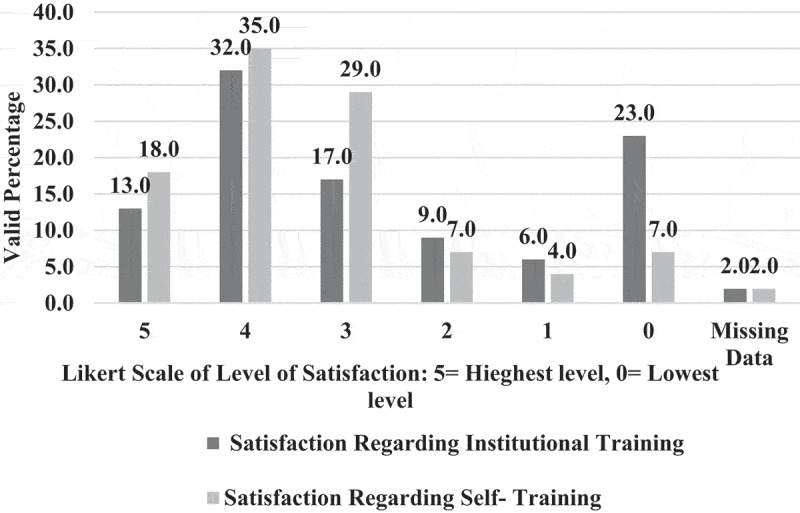
Satisfaction of the Egyptian Physical therapy Educators’ regarding online teaching training during the COVID-19, 2021 (N = 100).

**Figure 2.**
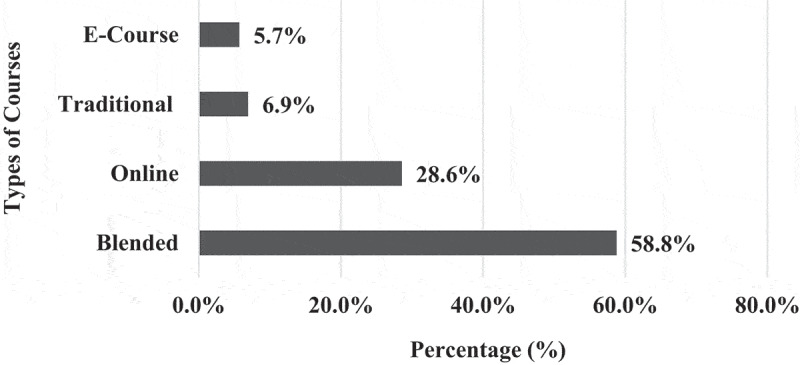
Frequency of use of the different types of courses during COVID-19 outbreak 2021 in Egyptian physical therapy colleges (N = 245 courses).

**Figure 3.**
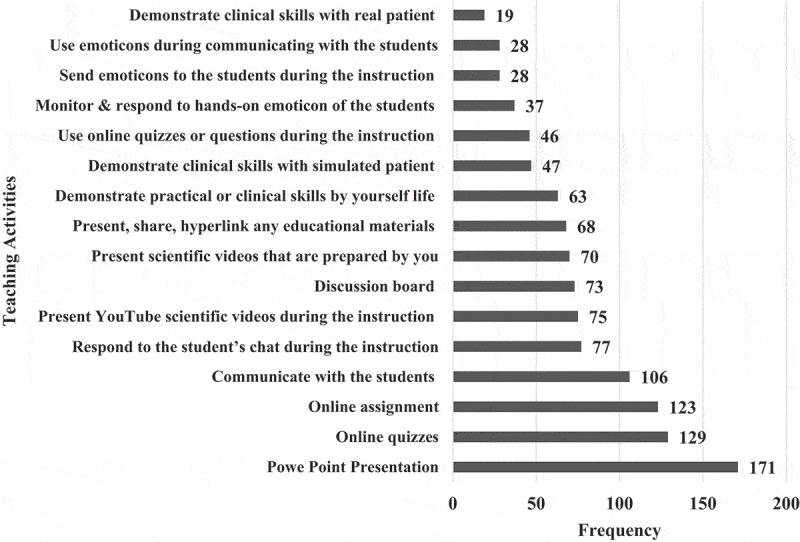
Frequency of used online teaching tools/activities by the Egyptian Physical therapy Educators during COVID-19 outbreak 2021.

**Figure 4.**
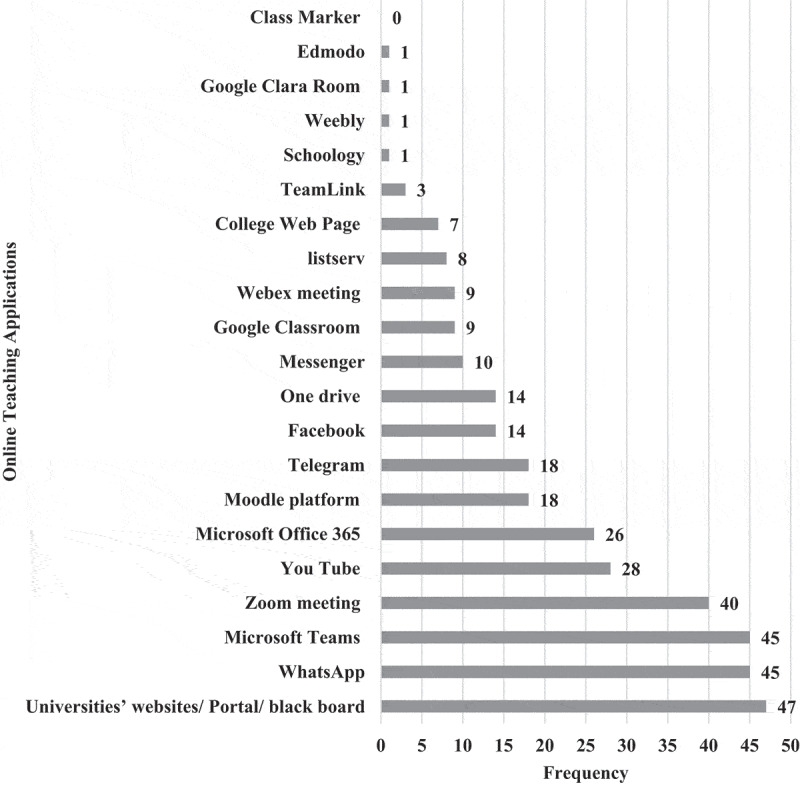
Frequency of used online teaching applications by the Egyptian Physical therapy Educators during COVID-19 outbreak 2021.

**1 – EPTEs OT profile during COVID-19 outbreak 2021**: As shown in Table 3, most (83.0%) of EPTEs did not use OT before the COVID-19 outbreak and for those who used it before, 58.8% of them had only 1 or 2 years of previous experience. Three-quarters (75.2%) of participants received institutional training (IT) (training introduced by the universities in form of workshops) on OT while the majority (93.0%) of them practiced self-training (ST) (training practiced independently by the educators themselves). The percentage of EPTEs who reported availability of institutional educational support centers for OT was higher than those who reported availability of college centers (78.7% and 54.1%) and they were more satisfied with ST than with IT (Figure 1). During the COVID-19 outbreak of spring semester – 2021, out of the 245 courses delivered by EPTEs, blended courses were the most frequent (58.8%) and they included (58.7 ± 9.4%) hours of OT (Figure 2). Table 4 shows that the most frequent practices of EPTEs were for theoretical instructions (40.0%), mixed OT approach (60.7%), OT from both work and home (58.0%), instructions scheduled outside working hours for from home OT (54.2%), and ‘the anytime style’ for communication with students (67.1%). PowerPoint presentations were the most frequently used OT tool (Figure 3) while university websites, Microsoft teams, and WhatsApp were the most frequently used OT applications (Figure 4).

**Table 3.** Practices of the Egyptian Physical Therapy Colleges and Educators during COVID-19 outbreak 2021

Participants’ use of online teaching before COVID-19 outbreak									
Use of online teaching				Frequency			Valid percentage (%)		
No previous use				83			83.00		
Previous use				17			17.0		
Total responses				100			100.0		
Missing data				2			2.0		
b. Availability of institutional training and practice of self-training on online teaching									
Institutional training	Frequency	Valid percentage (%)			Self-training	Frequency			Valid percentage (%)
Available	76	75.2			Practiced	93			93.0
Not available	25	24.8			Not practiced	7			7.0
Total responses	101	100.0			Total responses	100			100.0
Missing data	1	1.0			Missing data	2			2.0
c. Duration of institutional and self-training on online teaching									
Hours of training		Institutional training				Self-training			
Minimum		0				0			
Maximum		40				60			
Median		4				6			
Mode		0				2			
d. Availability of educational support center									
Availability of the educational support center/unit		Institutional				College			
Frequency		Valid percentage (%)				Frequency		Valid percentage (%)	
Available		78	78.7			53		54.1	
Not available		1	1.0			45		45.9	
I am not sure		20	20.2						
Total responses		99	100.0			98		100.0	
Missing data		3	2.9			4		3.9

**Table 4.** Egyptian Physical Therapy Educators’ profile regarding online teaching during COVID-19 outbreak 2021

Egyptian Physical Therapy Educators delivered types of instructions		
Instruction type	Frequency	Percentage (%)
Theoretical	74	40.0
Practical instruction	22	11.9
Clinical	5	2.7
Theoretical + Practical+ Clinical	27	14.6
Theoretical + Practical	33	17.8
Theoretical + Clinical	11	5.9
Practical + Clinical	13	7.0
Total	185	100.0
b. The used approaches of the online teaching		
Approach		
Synchronous	24	28.6
Asynchronous	9	10.7
Mixed	51	60.7
Total Responses	84	100.0
c. The placement of application of online teaching		
Placement		
Work	5	5.7
Home	32	36.4
Both	51	58.0
Total Responses	88	100.0
d. Schedule of the instruction for online teaching from home		
Instruction schedule		
During the usual working hours (9 am- 4 pm)	38	45.8
Outside the usual working hours (9 am- 4 pm)	45	54.2
Total responses	83	100.0
e. Time style of communication with the students		
Allowed communication time style		
Only during the scheduled instruction time	10	11.8
Outside the scheduled instruction time during the daytime but not nighttime	18	21.2
Any time	57	67.1
Total responses	85	100.0

**2 – Characteristics of EPTEs and their institutions that influenced OT profile during COVID-19 outbreak 2021**: Juniors (1–9 years academic experience) EPTEs were positively associated with previous use of OT (N = 100, P = 0.033) (Table 5A). Results showed that there were no associations between the following characters and previous OT use, university sector (N = 101, P = 0.277), EPTEs’ rank (N = 100, P = 0.751), subspecialty (N = 100, P = 0.375), and college commence years (N = 99, P = 0.522). Private universities showed a significant advantage over public universities regarding the provision of IT (N = 101, P = 0.003) and availability of institutional educational support centers (N = 99, P = 0.0001) (Table 5B, and C), while there were no significant differences regarding ST (N = 101, P = 0.715) or college center (N = 99, P = 0.186).

**Table 5.** Egyptian Physical Therapy Educators/theirinstitutions’ characteristics of positive association with online teaching during COVID-19 outbreak

a. Previous use of online teaching and Egyptian Physical Therapy Educators’ academic experience (N = 100), (P = 0.033)					
Academic experience (Years)			Previous use of online teaching		Total
			No previous use	Previous use	
1–9	Count (Raw %)		43 (82.7%)	9 (17.3%)	52.0 (100.0%)
10–19	Count (Raw %)		21 (87.5%)	3 (12.5%)	24 (100.0%)
20–29	Count (Raw %)		15 (88.2%)	2 (11.8%)	17 (100.0%)
30–39	Count (Raw %)		4 (80.0%)	1 (20.0%)	5 (100.0%)
40–49	Count (Raw %)		0 (0.0%)	2 (100.0%)	2 (100.0%)
Total	Count (Raw %)		83 (83.0%)	17 (17.0%)	100 (100.0%)
b. The university sector and the provision of institutional training (N = 101), (P = 0.003)					
University sector			Provision of institutional training		Total
			No	Yes	
Public		Count (Row %)	19 (37.3%)	32 (62.8%)	51 (100.0%)
Private		Count (Row %)	6 (12.0%)	44 (88.0%)	50 (100.0%)
Total		Count (Row %)	25 (24.8%)	76 (75.2%)	101 (100.0%)

Majority of EPTEs in public (92.2%) and private (94.0%) universities practiced ST with no significant difference (N = 101, P = 0.715) and with no influence of their rank (N = 100, P = 0.647) or their academic experience (N = 100, P = 0.956). Also, there was no association between training hours of either institutional (N = 99, P = 0.095) or ST (N = 100, P = 0.610) and the university sector. Also, being a private or a public university proved no significant input regarding the level of EPTEs satisfaction either for IT (N = 100, P = 0.887) or for ST (N = 100, P = 0.098). In addition, university sector did not impact level of technical skills of EPTEs (N = 60, P = 0.450) or of students (N = 100, P = 0.452). Characters of EPTEs including their rank and academic experiences did not interfere with OT used approach (N = 58, P = 0.149 and N = 58, P = 0.820), or placement of application (N = 60, P = 0.379 and N = 60, P = 0.816), or instruction schedule style (N = 56, P = 0.492 and N = 56, P = 0.153), or even communication style (N = 58, P = 0.800 and N = 58, P = 0.090). Also, university sector showed no association with any of the above-mentioned variables of OT (N = 58, P = 0.600 for approach, N = 60, P = 0.076 for placement, N = 60, P = 0.086 for instruction schedule, and N = 58, P = 0.859 for communication style). It is to be noted that the university sector did not influence the most used application (N = 57, P = 0.279) and that being diseased with COVID-19 did not influence the placement of application of OT (N = 60, P = 0.563). Academic rank significantly (N = 57, P = 0.006) was associated with the most used application as most (63.8%) of users of university websites were full or associate professors, and 53.3% of users of Microsoft teams were lecturers. Also, most used applications showed significant (N = 57, P = 0.020) association with EPTEs’ years of academic experience as (47.4%) of the users of university websites were with 20–29 years of experience and (57.9%) of users of Microsoft teams and (71.4%) of users of Zoom meeting were with 1–9 years of experience. No association was found between educators’ technical skills and their rank (N = 60, P = 0.139) or their years of experience (N = 60, P = 0.940).

**3 – EPTEs’ perceptions regarding the usefulness of OT for Physical therapy colleges, advantages, and disadvantages**

**Figure 5.**
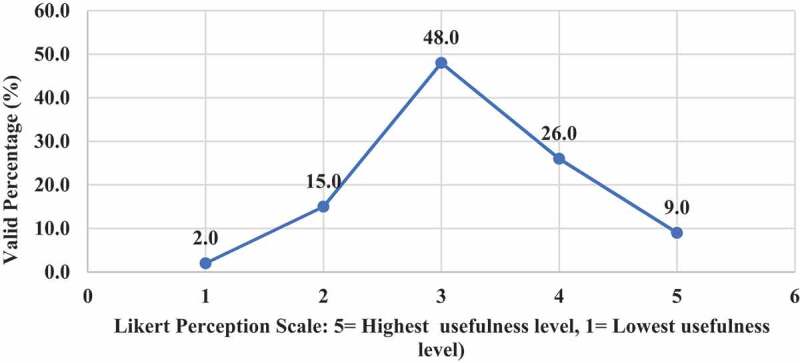
Perception of the Egyptian Physical therapy Educators regarding online teaching usefulness to physical therapy colleges (N = 100).

**Figure 6.**
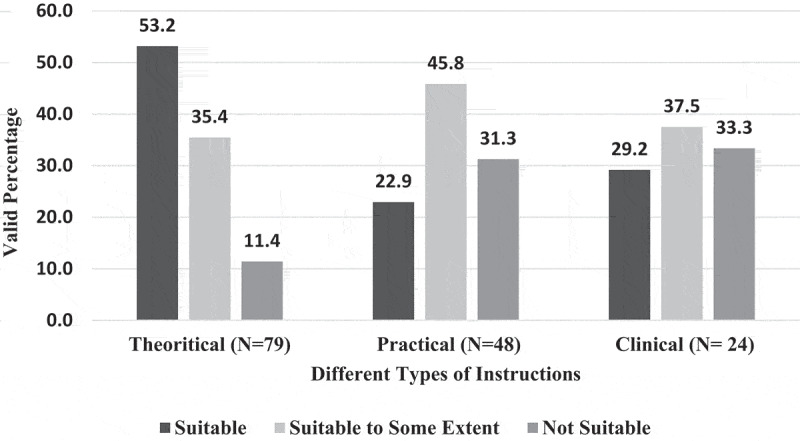
Perception of the Egyptian Physical therapy Educators regarding online teaching suitableness to instruction’s types during COVID-19 outbreak.

**Figure 7.**
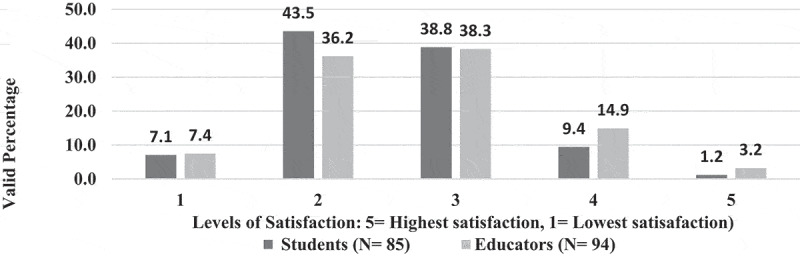
Satisfaction of the Egyptian Physical therapy Educators and students regarding quality of the delivered courses during COVID-19 outbreak.

Only 9% of EPTEs perceived (Likert scale 5) useful essence of OT for PT colleges (Figure 5) and they showed the least level of positive attitude toward usefulness of OT for practical instructions (22.9%), Figure 6. There was no association between EPTEs’ perception regarding usefulness of OT and their university sector (N = 100, P = 0.789) or colleges commence year (N = 98, P = 0.813), ‘ rank (N = 98, P = 0.847), years of experience (N = 99, P = 0.839), previous use of online teaching (N = 100, P = 0.803), or self-efficacy (N = 100, P = 0.663). Figure 7 presents levels of satisfaction of both EPTEs and their students regarding the teaching quality of delivered courses.

**Figure 8.**
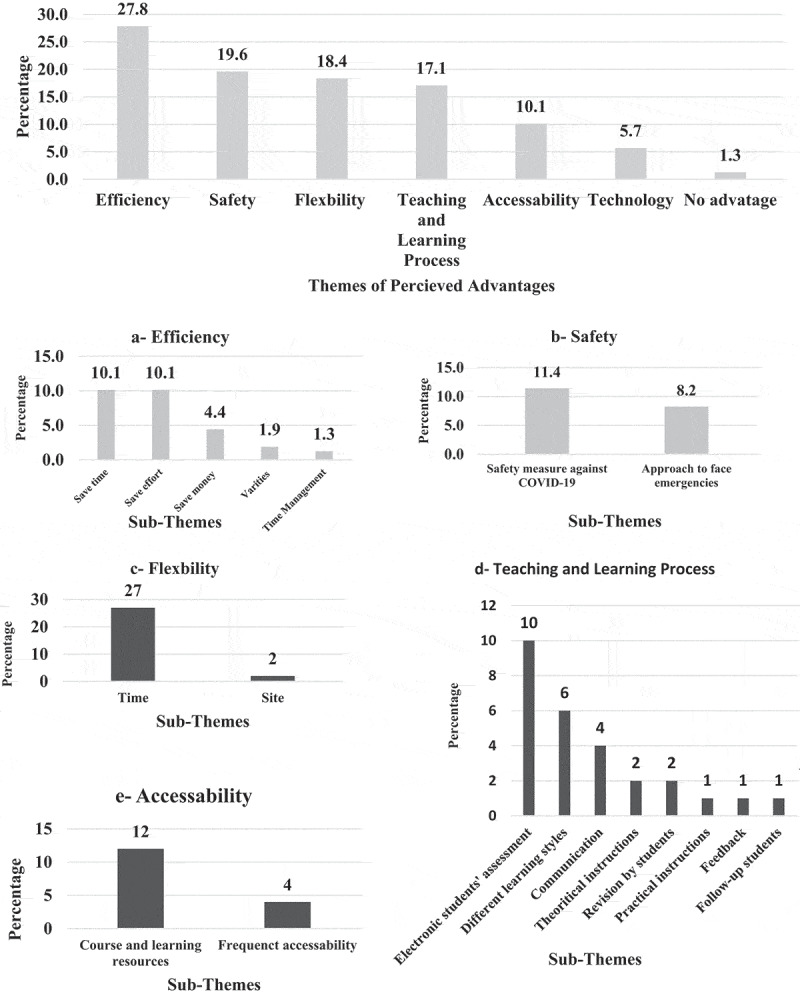
Perception of the Egyptian Physical therapy Educators Regarding the online teaching advantages during COVID-19 outbreak (N = 158 responses).

**Figure 9.**
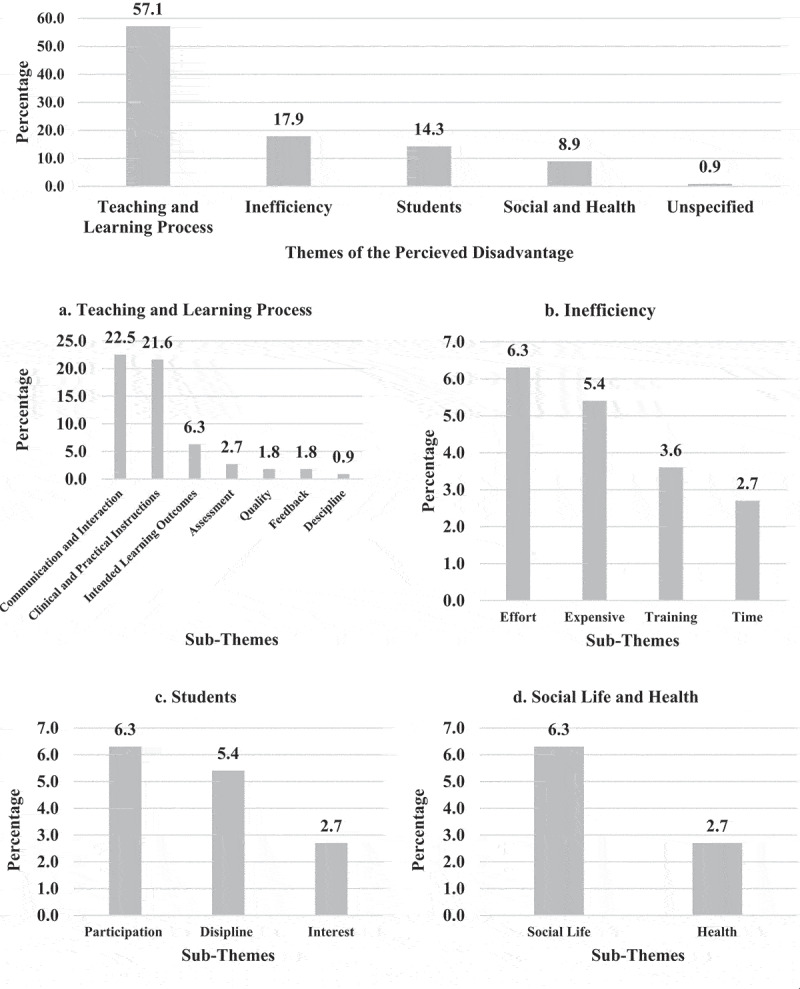
Perception of the Egyptian Physical therapy Educators regarding the online teaching disadvantages during COVID-19 outbreak (N = 111 responses).

EPTEs had 158 responses regarding advantages versus 111 responses regarding disadvantages of OT for PT colleges. Themes and sub-themes of advantages and disadvantages are presented in Figure 8 and Figure 9.

**Figure 10.**
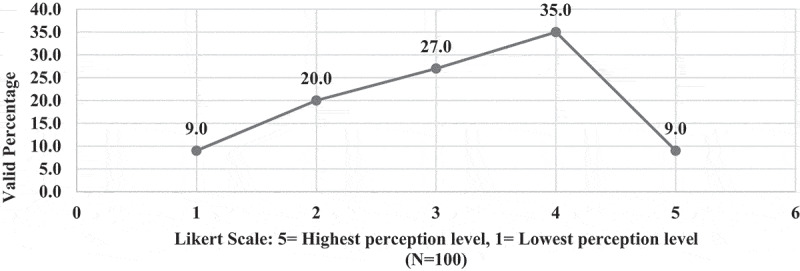
Perception of the Egyptian Physical Therapy Educators regarding their colleges readiness for online teaching during COVID-19 outbreak (N = 100 responses).

**Figure 11.**
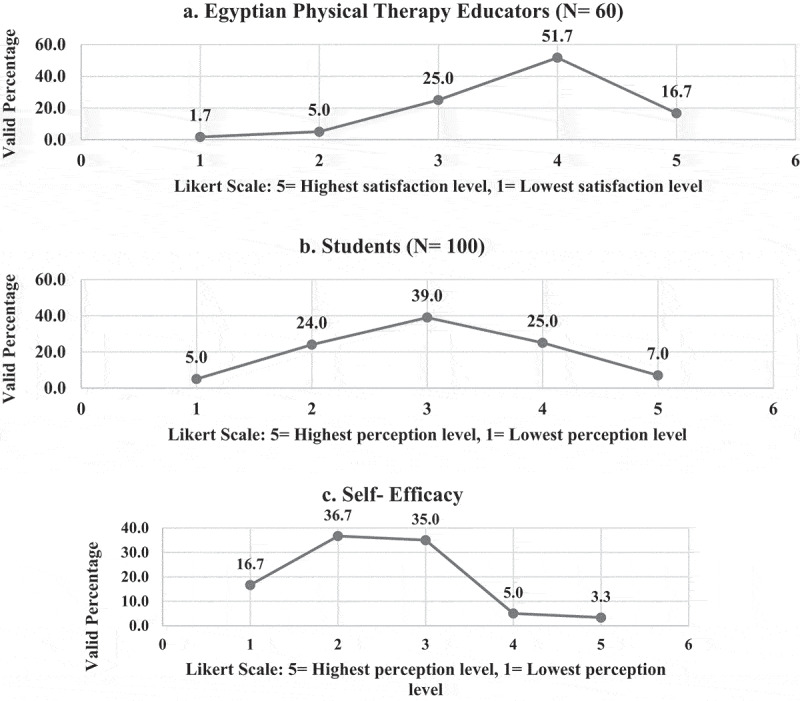
Perception of the Egyptian Physical Therapy Educators regarding their students technical skills and their self-efficacy during COVID-19 outbreak.

**Figure 12.**
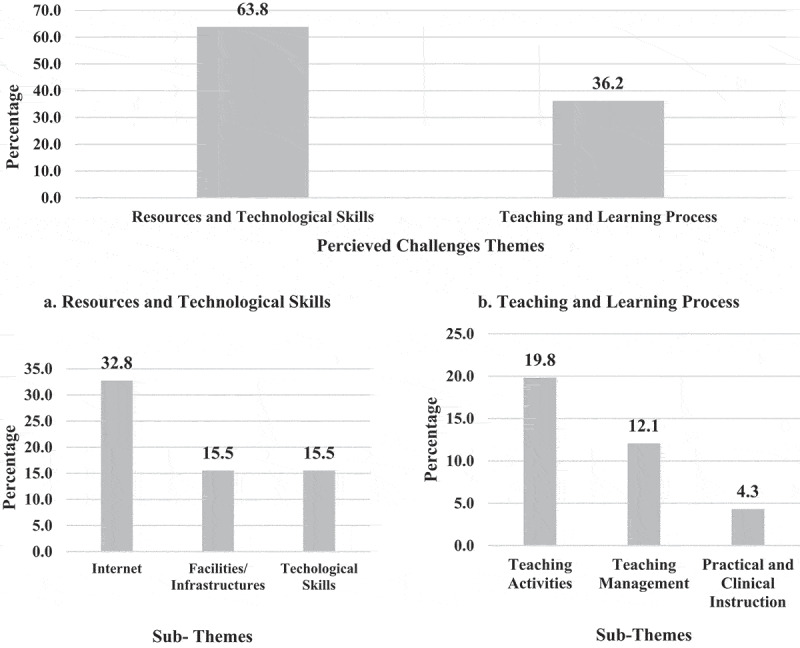
Perception of the Egyptian Physical therapy Educators regarding online teaching challenges during COVID-19 outbreak (N = 116 responses).

**Figure 13.**
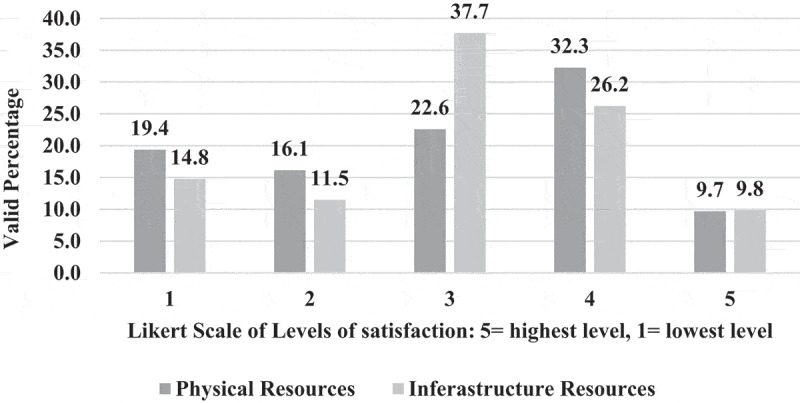
Satisfaction of the Egyptian Physical Therapy Educators regarding college’s support of physical/infrastructure resources during COVID-19 outbreak.

**4 – Readiness of Egyptian physical therapy colleges and educators to the application of OT and challenges**: Less than one-tenth (9%) of EPTEs showed the highest positive attitude (Likert scale 5) toward readiness of their colleges to the application of OT in terms of availability of supporting infrastructure and resources (Figure 10). While (68.4%) of EPTEs were satisfied with their technological skills (Likert scale 5 and 4), (32%) of them revealed positive attitudes toward their students’ technological skills, and (8.3%) of them had perceived self-efficacy Figure 11. The EPTEs’ perceived challenges presented in Figure 12 and Figure 13 declare that EPTEs’ satisfaction (Likert scale 5) with supplied physical resources and infrastructures by their colleges were 9.7% and 9.8%, respectively.

**Figure 14.**
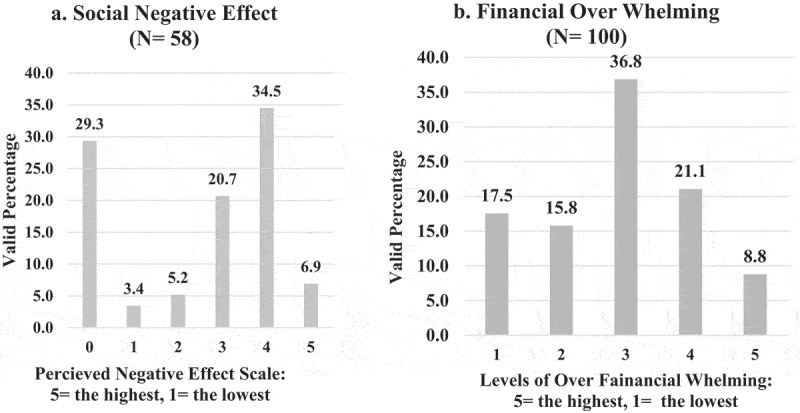
Social negative effect and financial over whelming of the Egyptian Physical therapy Educators by the online teaching from home during COVID-19 outbreak.

**5 – Social and financial implications on EPTEs caused by OT from home during the COVID-19 outbreak**: Two-thirds (68%) of EPTEs reported a negative effect of OT on their social life and (29.9%) of them reported high levels of financial overwhelming (Figure 14). One EPTE reported that he/she received facility support (internet–laptop/computer) for OT from home (N = 59). Results showed that lower rank educators were associated with a higher perception of negative social impact (N = 58, P = 0.027).

**Figure 15.**
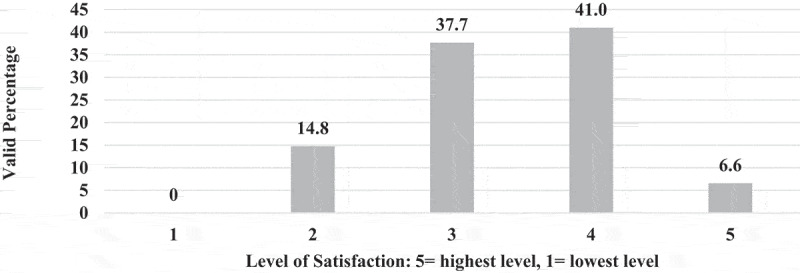
The overall satisfaction of the Egyptian Physical Therapy Educators regarding the online teaching during COVID-19 outbreak 2021 (N = 61).

**6 – Overall satisfaction and associated institutional and educators’ characters**: Less than half (47.6%) of EPTEs are overall satisfied (Likert scale 4 and 5) with OT experience (Figure 15). Associated characters included their perceived level of effectiveness of ST (N = 60, P = 0.033), and perceived level of readiness of their colleges (N = 59, P = 0.032). Characters showed no association include EPTEs’ years of academic experience (N = 60, P = 0.464), university sector (N = 60, P = 0.103), educators’ previous use of OT (N = 60, P = 0.243), perception regarding effectiveness of IT (N = P = 0.124), usefulness of OT (N = 59, P = 0.054), educators’ technical skills (N = 57, P = 0.380), number of total taught courses (N = 59, P = 0.448), percentage of OT for blended courses (N = 56, P = 0.778), the placement of OT (N = 56, P = 0.154), style of communication with students (N = 55, 0.382), perception of over whelming (N = 54, P = 0.050), and level of negative impact on social life (N = 55, P = 0.061).

## Discussion

This study aimed to answer questions related to the OT in Egyptian PT colleges during the COVID-19 outbreak in, the Spring semester of 2021. Results of EPTEs’ OT profile raised a hypothesis that it is not only their previous experience was limited, but also their experience during the spring semester of 2021 was primitive. This hypothesis is based on three reasons, the first one is the specification of PT programs in which a major part is related to practical and clinical skills and most PT courses include a theoretical part as well as practical and/or clinical parts that require face-to-face communication between educators, students, and patients. Specification of PT program might also be responsible for blended teaching being the most used approach. Through the online portion of blended teaching, educators could deliver the course’s theoretical part and in the face-to-face traditional portion, they could deliver practical and clinical parts. Meanwhile, OT was the second most used approach, and this could be explained by the fact that PT programs include a remarkable number of theoretical courses which can fit with OT. EPTEs emphasized the effect of PT program specification on other different occasions including their perceptions regarding the advantages, disadvantages, and challenges of OT. When the EPTEs perceived the teaching and learning process as an advantage theme of OT they declared that it was suitable mainly for online students’ assessment and none of them found it advantageous for clinical instructions. The positive perception of the EPTEs regarding the advantage of online students’ assessment is supported by the results of the study done by Rossettini et al. in 2021 in which a comparison of Italian PT students’ performance in an oral exam for a case-based course between two groups of students (online and face-to-face) was for the advantage of the online group [26]. But it should be considered that the higher performance in the oral exam for the online group of students might not actually represent higher knowledge and ability to acquire this knowledge in a clinical scenario as claimed by the researchers. This is because this higher performance might be related to the get rid of the tension or stress offered by the online oral exam which is not a case guaranteed by the face-to-face oral exam. But in real clinical practice, the students should be able to face and manage these stresses that is why on many occasions, items of the oral exam rubrics is related to the student’s confidence and stress management. The study of Huhn et al. (2013) compared two different teaching modalities: virtual (in an on-campus computer lab with an educator available to answer questions) and live (a large group discussion with an educator facilitator) showed no differences in the students’ clinical reasoning, knowledge acquisition, and transfer of knowledge [32]. The no difference between virtual and live teaching might be because even in virtual teaching, the educator is present with the students in a face-to-face condition. It can be said that this finding is supporting the EPTEs’ negative perception of the OT for clinical and practical instructions and is emphasizing the importance of face-to-face teaching for knowledge and skill acquisition. Meanwhile, when the EPTEs perceived the teaching and learning process as a disadvantage theme of OT, they stated that ‘OT offered poor interaction and communication with students and consequently negatively affected practical and clinical instructions’. Add to that, ‘difficulty to prepare materials and activities fitting to objectives of practical and clinical instructions and at the same time applicable for OT’ was among the challenges stated by EPTEs. Other studies [33,34] reported the insufficiency of interactive material as one of the barriers to OT, particularly websites. Students’ perceived satisfaction was different from that of the EPTEs, the Italian online and face-to-face two-group students [26] showed equal satisfaction with the case-based course introduced to the two groups. The difference in perception between EPTEs and the Italian PT students may be related to the difference between the case-based course and the clinical and practical courses. EPTEs showed a neutral perception of the usefulness of OT for PT colleges and at the same time the order of their positive attitude toward the suitableness of different types of instructions was first for the theoretical instructions, followed by the practical and then the clinical instructions. So, their neutral perception regarding usefulness can be explained as they partly perceived OT as useful (for the theoretical instructions) and partly not useful (for practical and clinical instructions). And it is to be noted that the absence of any association between educators’ perception and their rank, years of experience, self-efficacy, university sector, or college commence year empowers the role of the program specification. Moreover, both the educators and their students showed marked dissatisfaction with the quality of teaching of delivered courses through OT and this result digs more into the hypothesis that PT program specification indicates that OT is not the perfect teaching approach for PT colleges.

The second claimed reason for the limited and primitive experience of EPTEs on the OT is the shortage of financial and physical resources. Although technology recently introduced into health professions education, including PT, and tools like simulated and virtual reality labs are used in some well-resourced PT colleges in some developed countries, the insufficiency of resources made traditional teaching the convenient, economical teaching approach for Egyptian PT colleges. On the other hand, even in the developed countries, Aleksandra highlighted the need for careful planning for the incorporation of technology into learning and teaching practices despite the technology having a place in health professions education [11]. Anyway, the inefficacy of OT was perceived by EPTEs as the second disadvantage because they had to use their personal computer or laptop and their source of the internet when they used OT from home as their colleges did not support them with any computers/laptops or internet access. That is why about one-third of educators perceived that OT from home financially overwhelmed them.

The modesty and variability of technological skills of EPTEs are the third claimed reason. A common observation is that many of the senior EPTEs are not convenient with technology use even the simplest ones and this observation is supported by the current study results as more than half of EPTEs who previously used OT were, juniors, with less than 10 years of academic experience. Despite both the traditional approach, which might indicate no technical skills, and the e-courses approach, which with no doubt indicates the highest technical skills, was the least used, this result highlights the variability of EPTEs’ technical skills. On one hand, the PT program includes no or few courses, which are only practical or clinical courses that require traditional teaching, so most probably the used traditional teaching was indicating the personal choice of some of the senior EPTEs for their preference. And on the other hand, the e-course approach might be the selection for a minority of EPTEs who had previous experience with OT. It is to be noted that using the university website in which all the required was uploading course material including voice record PowerPoint presentations was preferred by an associate or full professor EPTEs of 20–29 years of experience, while Microsoft teams and Zoom meeting, which implies more interaction with the students and more technological skills were preferred by lecturers of 1–9 years of experience. This note provides another piece of evidence for the variability in technological skills for the advantages of the juniors who have previous experience with OT. One of the teaching activities which requires interaction is the discussion board, which was moderately used by the EPTEs, and which was proved by other studies [34,35] to provide significant improvement in knowledge acquisition (measured by students’ final marks).

Although results showed a higher perception of EPTEs to their technical skills as well as no association between EPTEs’ technical skills and neither their rank nor their academic experience, these results are seemed to be based on educators’ perceptions while the above-mentioned evidence indicates different real-life practices. Add to that the educators themselves showed poor self-efficacy, which contradicts their perception regarding their technical skills. Moreover, deficiency of technical skills was one of the marks reported as a challenge.

During the outbreak, the shift into OT was managed through different aspects including training on OT and it was remarkable that the percentage of EPTEs who practiced ST was more than those who received IT and that they perceived more effectiveness for ST than IT. This result can be explained by a claimed concept that during a crisis, personal actions might be faster and more responsive, and more flexible than institutional ones. Also, it can be said that EPTEs are skillful in the English language, which is the study language of PT programs, and this facilitated their use of the available manuals and tutorial videos to train themselves on applications and tools that were suitable for their instructions, friendly use, or more familiar with, that is why they perceived more satisfaction with their ST than IT. On the other side, IT needs approvals, preparation of materials, schedule programs, announcements, and train-the-trainers. University website, Microsoft Teams, and WhatsApp were the first three most used applications by EPTES. If IT included these tools, the educators would be satisfied with IT and would not even need for ST or at least would not need to practice ST for a longer time than the IT, but results revealed that this was not the case. Most probably the IT included only training on the university website as it is the official platform of the universities and it already established even before the outbreak, as a fulfillment of the accreditation requirements, and EPTEs might receive a kind of instruction to use it and that is why it was number one used tool. At the same time, it looks like the websites were not sufficient for educators, and they train themselves on other applications such as Microsoft Teams and WhatsApp, so it was logical to be the second and third most used after the university website. Although the website was not totally satisfying to EPTEs, other studies [30,35,36] showed an improvement in practical skills in groups of students using websites, but this could be because these websites included web-based tutorials or online repositories with videos and patient-therapist simulations. Also, it is to be noted that the pattern of use of applications supported that EPTEs’ experience with OT was primitive during the outbreak of 2021, and most probably was dependent on personal trial and preference. For example, an application for social communication like WhatsApp was in the second order of use even before the Microsoft Teams application while applications like Schoology, Google Classroom, and Edmodo which are more professional for OT were least frequently used.

The management of the COVID-19 outbreak also included the provision of institutional and educational support centers, but it could be assumed that the shortage of resources and modest experience that affected the EPTEs profile of OT also had an extended effect on these centers and influenced its effectiveness that is why the most common used OT teaching activity was the PowerPoint presentation, just like in traditional teaching. Another reason that could explain the massive use of PowerPoint presentations is that most of the participated EPTEs were lecturers, associates, and full professors who were responsible for theoretical instructions, which were also the most delivered type of instructions. But the effectiveness of educational support centers is still questionable as one of the challenges reported by EPTEs was a scarcity of facilities in the centers during the preparation of scientific videos for clinical and practical skills and that they had to prepare these videos by themselves. Despite the moderate reported effectiveness of IT and educational support centers, results showed that private universities, which are famous with higher resources than many public universities, had the advantage over public universities regarding the provision of IT and availability of educational support centers, which echo the importance of resources for OT implementation.

The qualitative analysis showed that EPTE’s experience with the OT during the COVID-19 outbreak in 2021 perceived groups of advantages, disadvantages, and challenges. The efficiency of OT was the first most perceived advantage as the educators said that ‘OT saved time and money mainly those spent in transportation, especially for students’. As if OT was a solution for a huge number of Egyptian populations, including university students, and as stated by EPTEs, ‘cost of transportation deducts a considerable percentage of the family income especially if the students’ university was in a state away from their residence’. This is like what El Gamal stated that the increased flexibility with OT may provide a solution for Egyptian students who may be deprived of higher education because of physical limitations to reach universities that are usually located in central cities in Egypt [14]. But at the same time, EPTEs perceived OT as inefficient and expressed that ‘time was consumed to prepare educational material in a form suitable for OT’ and while PowerPoint presentations its place as the most common teaching activity as mentioned earlier, educators needed to apply voice record on these presentations to upload them on websites or send them to their students. Also, educators consumed time preparing online quizzes and some of them prepared scientific videos for practical instructions. Although EPTEs perceived safety as a second advantage of OT, they stressed that it is only for emergencies and crises to overcome certain pandemics such as COVID-19 but not in normal situations. Also, EPTEs found that OT offered them great freedom and they reported flexibility as the third advantage. This result provides an explanation for their OT practice pattern as the highest percentage of them used OT from both home and work, with a mixed synchronous and asynchronous approach, scheduled instructions outside the regular working hours, and even allowed their students to communicate them at any time as if they were excited with the first-time use of OT and with teaching outside campus with its rigorous schedule and other academic rules. This pattern provides proof to the above-mentioned concept that it was not only limited to previous experience but also that the experience during the COVID-19 outbreak in 2021 was primitive. The disadvantage also included the health aspect as the educator stated that ‘sitting position for a long time in front of the screen of laptop/or computer to prepare or to apply OT was harmful’.

The impact of OT on EPTEs’ social life was one of the perceived disadvantages of OT, more than two-thirds of them reported a negative impact on their social life, and (41.4%) of them rated the highest scales (4 and 5) for this negative impact of OT when practiced from home. Educators stated that ‘OT consumed their family time’. But it was remarkable to find that a close percent (46.2%) of educators rated the lowest scales (0, 1, and 2) and this can be explained by the result, which showed an association between social negative impact and rank of educators as most of the educators who perceived high impact level were lectures, while those perceived low impacts were associate or full professor. The previous assumption that lecturers were with higher technical skills and applied more interactive applications on OT, which required more time and effort for preparation consequently affected lecturers’ social life while professors were mostly those who used university websites and just uploaded their presentations or were the most traditional teaching users, so they perceived lower social negative impact. Add to that, lecturers have a teaching load more than professors, and might their younger age indicate more family responsibilities than professors. This negative social effect of OT was also reported in a study done in three countries (Brazil, Cyprus, and the USA) in which it was found that the PT educators faced a challenge to manage the work–life balance [3].

This study showed that only less than one-tenth of EPTEs had full trust in the readiness of their colleges to apply OT and rated scale (5) and even those who rated (4) were another one-third of educators. These results were logical because readiness was perceived according to the availability of supporting infrastructure and resources and all educators, except one, reported that they were supported with neither laptop/computer nor internet access when they applied the OT from home. Echo the limited effect of IT and the poor facilities in educational support centers add proof to this result and provide an explanation to the reported disadvantage that OT was inefficient and costly for educators and to the fact that shortage of resources and limited technological skills were the first challenge reported by EPTEs. On the other hand, it is to be noted that in other countries where the resources are not a problem, the PT educators reported challenges in applying OT for PT programs as challenges in making authentic connections with students, adapting to technological interruptions, and assessment of the students [3]. A systematic analysis of the changes in the universities’ teaching activities for adaptation of OT during the COVID-19 agreed with the current study challenges in terms of the lack of educators’ basic skills for OT, which make the creation of an online course a major challenge that caused frustration feelings to the interviewed educators [37].

Academics were then tasked with creating an entire online course overnight and found it vastly challenging. Many interviewees in this study recalled feeling frustrated and apprehensive at this moment, mainly due to their lack of basic skills for online teaching.

Complaints of EPTEs regarding difficulty with the internet in terms of speed and interruption are reported by other studies [28,29]. So, no wonder to find that educators’ satisfaction regarding available physical resources and infrastructures was very minor. Generally, EPTEs showed limited overall satisfaction with OT as less than half of them were satisfied with their experience with OT, and the association between their satisfaction level and their perception regarding their ST and their colleges’ readiness indicates the importance of the development of technical skills and PT colleges’ resources for OT.

## Conclusion

This study indicated that the EPTEs’ profile of OT was limited before COVID-19 and primitive during the outbreak 2021 and that OT is not the perfect teaching approach for PT colleges. Three reasons were claimed to be responsible for this profile including PT program specification, shortage of resources, and the modesty and variability of the educators’ technical skills. Different educators and institutional characters were associated with this profile and EPTEs perceived different advantages, disadvantages, and challenges. They also reported different levels of negative social impact by practicing OT from home as well as levels of financial overwhelming. EPTEs stated that their Egyptian PT colleges are not ready for OT.

## Limitation of the study

Analysis of the qualitative responses was performed by the author on an individual basis. The author in a solo task reviewed the responses and divided them into themes and sub-themes. No expert team or steering group was used.

## Data Availability

The datasets during and/or analyzed during the current study are available from the corresponding author on reasonable request.

## Abbreviations

OT, online teaching; PT, physical therapy; EPTEs, Egyptian Physical therapy Educators; IT, institutional training; ST, self-training.
